# Supplementary material for: Role of glycosylation mutations at the N-terminal domain of SARS-CoV-2 XEC variant in immune evasion, cell-cell fusion, and spike stability
Source: J Virol. 2025 Mar 26;99(4):e00242-25. doi: 10.1128/jvi.00242-25 (PMC11998534; doi:10.1128/jvi.00242-25)
Supplement: Fig. S1 — Antigenic distance units to variants of interest relative to D614G or JN.1 (related to Fig. 3). [file jvi.00242-25-s0001.pdf]

A

Bivalent HCWs			
AD (D614G)		AD (JN.1)	
JN.1	4.9	D614G	4.9
KP.3	5.3	KP.3	1.0
KP.3_T22N	5.4	KP.3_T22N	1.3
KP.3_F59S	6.8	KP.3_F59S	3.6
XEC	7.5	XEC	3.3
XEC_S24A	6.7	XEC_S24A	4.8
KP.3.1.1	7.3	KP.3.1.1	5.0
KP.3.1.1_T33A	6.1	KP.3.1.1_T33A	1.2

B

BA.2.86/JN.1-wave patients			
AD (D614G)		AD (JN.1)	
JN.1	3.4	D614G	3.4
KP.3	4.1	KP.3	1.7
KP.3_T22N	4.3	KP.3_T22N	1.7
KP.3_F59S	4.9	KP.3_F59S	3.1
XEC	5.7	XEC	3.4
XEC_S24A	5.2	XEC_S24A	4.2
KP.3.1.1	5.3	KP.3.1.1	2.9
KP.3.1.1_T33A	4.9	KP.3.1.1_T33A	1.8

C

XBB.1.5-monovalent hamsters			
AD (D614G)		AD (JN.1)	
JN.1	2.7	D614G	2.7
KP.3	2.7	KP.3	0.2
KP.3_T22N	2.8	KP.3_T22N	0.2
KP.3_F59S	2.5	KP.3_F59S	0.7
XEC	3.0	XEC	0.7
XEC_S24A	3.0	XEC_S24A	0.5
KP.3.1.1	2.7	KP.3.1.1	0.6
KP.3.1.1_T33A	2.8	KP.3.1.1_T33A	0.7

Fig S1: Antigenic distance units to variants of interest relative to D614G or JN.1 (Related to Fig. 3). Antigenic distance (AD) values were determined using Microsoft PowerPoint for each of the variants relative to D614G or JN.1 and listed for each cohort.
