# Supplementary material for: Role of glycosylation mutations at the N-terminal domain of SARS-CoV-2 XEC variant in immune evasion, cell-cell fusion, and spike stability
Source: J Virol. 2025 Mar 26;99(4):e00242-25. doi: 10.1128/jvi.00242-25 (PMC11998534; doi:10.1128/jvi.00242-25)
Supplement: Table S1 — Bivalent-vaccinated HCW and BA.2.86/JN.1-wave first responder cohorts. [file jvi.00242-25-s0002.pdf]

Table S1. Bivalent-vaccinated HCW and BA.2.86/JN.1-wave first responder cohorts

	**Bivalent Health Care Workers** (n=8)	**BA.2.86/JN.1 Wave Patients** (n=9)
**Age in Years at Sample Collection [Median (Range)]**	39 (27-46)	51 (34-77)
**Gender** [n (% of Total)]		
Male	5 (63%)	5 (56%)
Female	3 (37%)	4 (44%)
**Sample Collection Window**	Dec. 2022	Nov. 2023-Aug.2024
**Vaccine status** [n (% of Total)]	NA	
1-dose Pfizer	NA	1 (10%)
2-dose Moderna	NA	2 (20%)
4-dose Moderna	NA	1 (10%)
1-dose Moderna +1-dose Pfizer bivalent	NA	1 (10%)
1-dose Pfizer +1-dose Pfizer bivalent	NA	2(20%)
2-dose Pfizer +1-dose Pfizer bivalent	1 (12.5%)	NA
3-dose Pfizer +1-dose Moderna bivalent	NA	1 (14.3%)
3-dose Pfizer +1-dose Pfizer bivalent	3 (37.5%)	NA
3-dose Moderna +1-dose Moderna bivalent	4 (50%)	1 (14.3%)
Days from last vaccination	NA	621 (34-1033)
Days post the bivalent dose for recipients	65 (23-97)	NA
**COVID-19 positive** [n (% of Total)]	8 (80%)	9 (100%)
Days before sample collection [(Median Range)]	324 (182-994)	7 (1-10)
**Infected Variants**		
JN.1/BA.2.86	NA	2 (22%)
Undetermined	NA	8 (78%)

Summary of the demographic information for two cohorts used for neutralization experiments depicted in Figure 2. “NA” means the category is not applicable to the cohort.
